# Inferior vena cava calcification, a possible link with recurrent deep venous thrombosis and pulmonary embolism: a case study and review of literature

**DOI:** 10.1259/bjrcr.20180018

**Published:** 2018-04-16

**Authors:** Ahmed KA Ahmed, Vanina Finocchi, Salah Al-Agib

**Affiliations:** Department of Radiology, Nobles Hospital, Douglas, Isle of Man

## Abstract

Inferior vena cava (IVC) anomalies have been reported to have an association with deep venous thrombosis of the lower limbs. It is, therefore, necessary to study the IVC in recurrent cases of unprovoked deep venous thrombosis (DVT) and/or pulmonary embolism (PE), where all other causes have been excluded. We report a case of a 65-year-old male, who had recurrent episodes of DVT in the past 5 years; some of which associated with PE of unknown cause. CT thorax abdomen and pelvis did not find an obvious cause for the DVT and/or PE, however, it did highlight a diffuse calcification of the IVC. Only a few cases of calcification of the IVC have been reported in literature, and a number of them have been associated with clot formation and PE. We speculate that, as in other anomalies of the IVC, calcification of the IVC might slow the blood flow, and thus predispose to DVT and/or PE. Our opinion is that in all cases of unexplained DVT and/or PE, a careful examination of the IVC should be performed. Furthermore, when this condition is present, other risk factors for hypercoagulability should be avoided and anticoagulant therapy should be considered.

## INTRODUCTION

Inferior vena cava (IVC) anomalies have been reported to have an association with deep venous Thrombosis (DVT). This association was made mainly with congenital abnormalities such as atresia^1–3^ or hypoplasia of the IVC.^4^ The probable common underlying pathogenetic mechanism is an abnormal flow in the IVC. Many authors suggest a dedicated study of the IVC in patients with recurrent unexplained DVT.^1, 4^

The calcification of the IVC is a rare finding, mostly asymptomatic and incidental, of unclear aetiology.^5, 6^ It has been associated with clot formation and pulmonary embolism (PE).^5^

We report a case of recurrent DVT and PE in a patient with extensive calcification of the IVC, and we speculate a relationship between this IVC abnormality and the recurrent DVT/PE. As in other causes of IVC anomalies, the irregular calcification causes a disturbance in the blood flow, and thus may be related to clot formation.

It is, therefore, important that in all cases of unexplained DVT and/or PE, the radiologist pays particular attention to the IVC, and consider a possible correlation with clot formation. Furthermore, in patients with diffuse calcifications of the IVC, preventive measures should be implemented, such as reducing other hypercoagulability factors, or in the case of DVT/PE, consider lifelong anticoagulation.

## Clinical presentation

A 65-year-old male presented with a DVT in his right leg while on anticoagulant therapy.

He had a history of recurrent DVT and PEs. In total, he was treated for PE on three occasions; the most recent being 5 years ago. CT of the thorax abdomen and pelvis (TAP) was performed at that time; however, the calcification of the IVC was not mentioned, possibly because this was regarded as insignificant.

The patient had another episode of DVT 1 month prior to admission in Nobles Hospital, which was treated with 2 weeks of clexane and then 110 mg of dabigatran. The reason for the reduced dose of dabigatran is unknown. He does not meet the age criteria, has no renal impairment, has no bleeding tendency and is not concurrently using verapamil.

He had recently arrived via a long haul flight from New Zealand. He presented to A&E with pain, swelling and redness in his right leg, with a decreased range of movement, but denied any chest pain, cough or haemoptysis.

## Imaging findings

An ultrasound Doppler was performed which confirmed an extensive DVT involving the external iliac vein, common femoral vein, saphenous femoral junction, the proximal portion of the long saphenous vein and the femoral vein. The veins appeared enlarged, with free floating hyperechoic endoluminal material, and they were not compressible. A few enlarged inguinal lymph nodes were found, which were possibly reactive. The popliteal vein showed normal flow with no signs of endoluminal thrombus. The Iliac vessels were not visualised.

A CT TAP was requested to investigate the possible cause of the recurring DVT (mainly to exclude malignancy). The exam was performed with a 64-slice GE CT and 0.3 mm multiplanar reconstructions were obtained. 130 ml of 300 mg I ml^–1^ Iohexol contrast was administered at a rate of 4 ml s^−1^. The thorax and abdomen were scanned in the arterial phase; additionally, the abdomen and pelvis were scanned in the portovenous phase.

**Figure 1.**
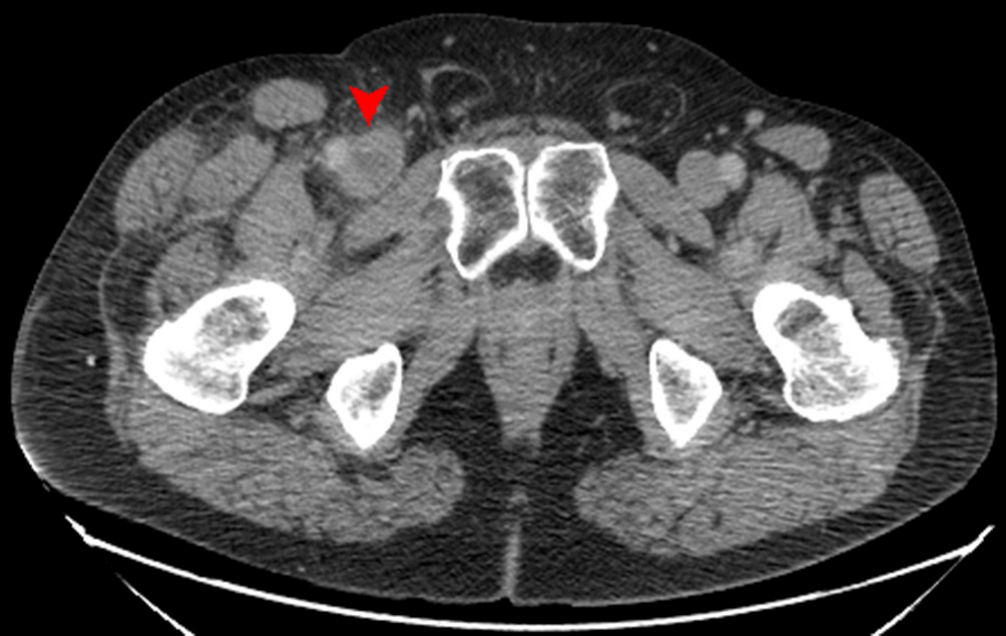
Axial CT scan showing a clot in the common femoral vein.

The CT TAP confirmed the presence and extension of the clot (Figure 1). No signs of malignancy or other clear cause of DVT were found.

**Figure 2.**
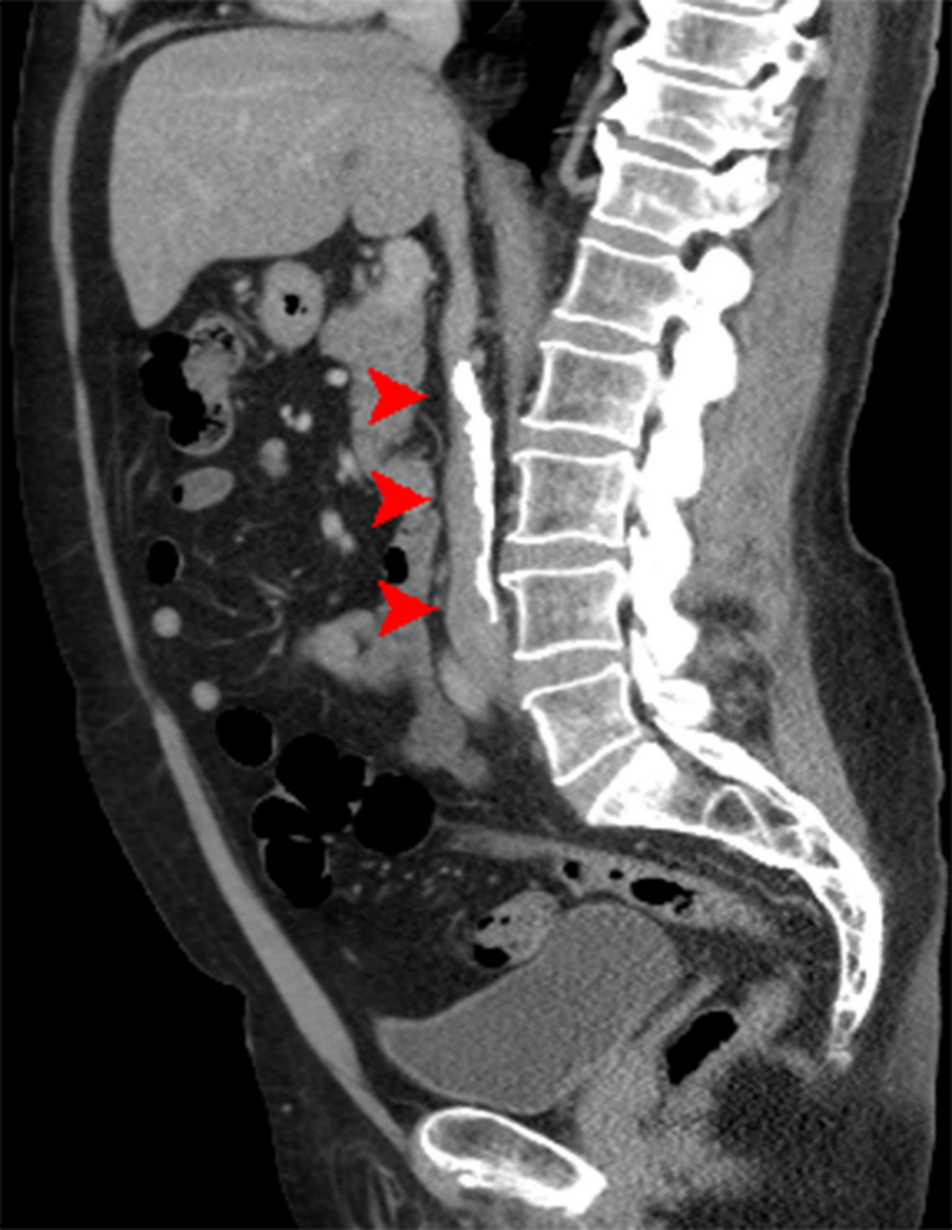
Sagittal CT showing calcification of the IVC. IVC, inferi orvena cava.

**Figure 3.**
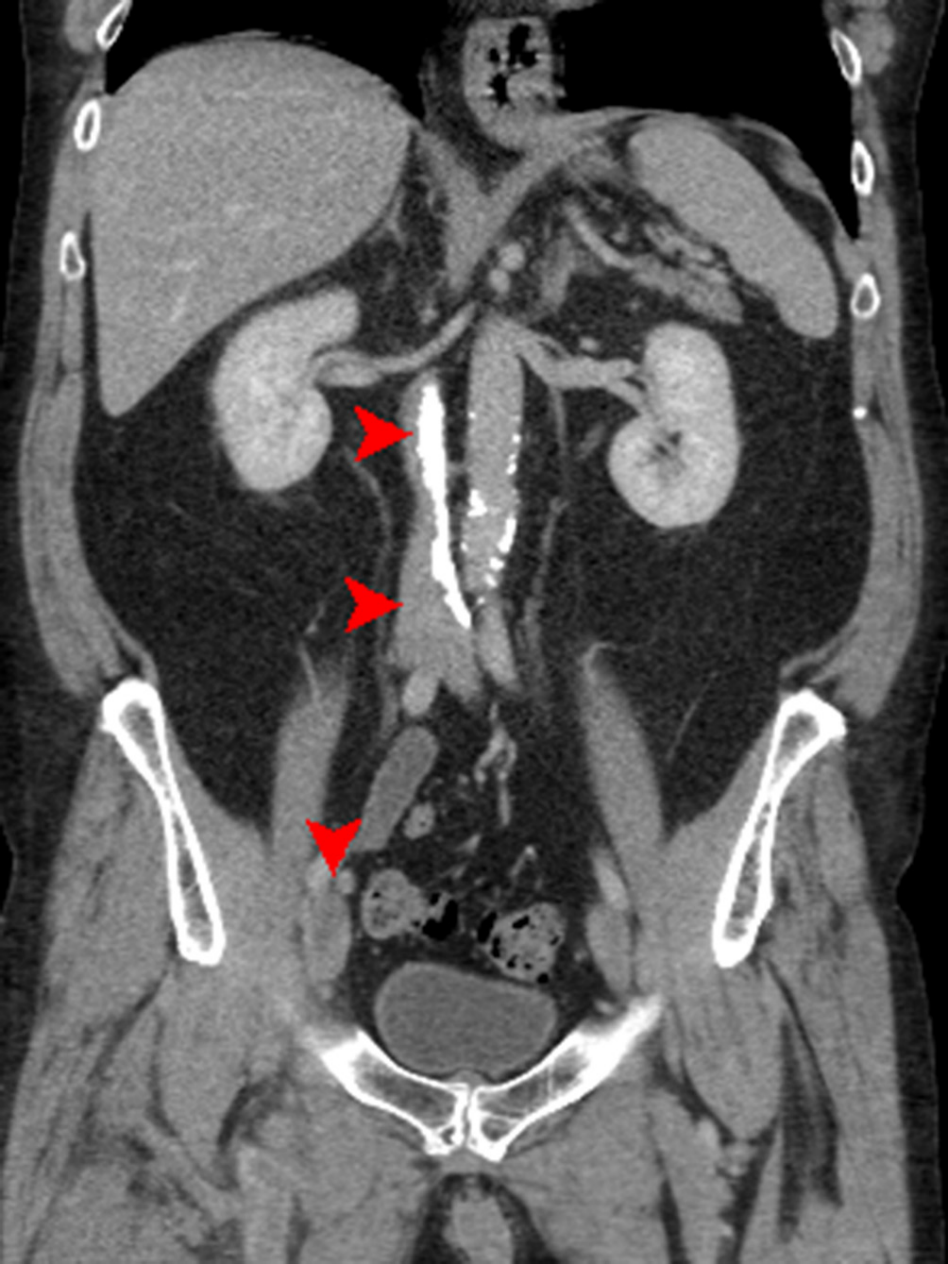
Coronal CT showing calcification of the IVC and thrombus in the terminal iliac/common femoral vein. IVC, inferior vena cava.

**Figure 4.**
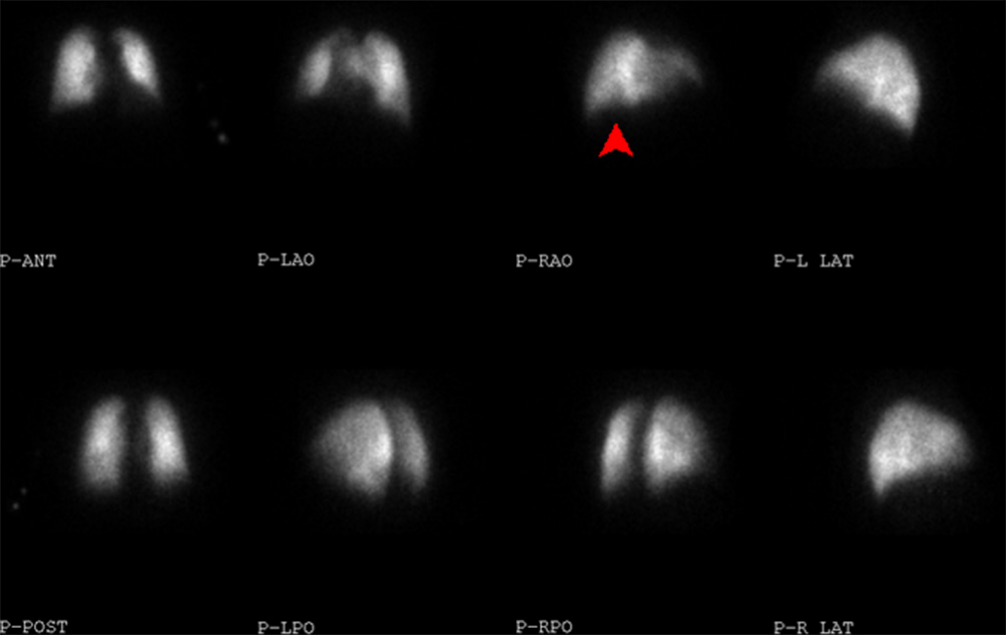
Lung perfusion scan showing a single small subsegmental perfusion defect.

The exam, however, showed significant and diffuse calcification of the IVC, with an extension of approximately 10 cm, and extended from the entry point of the left renal vein until the confluence of the common iliac veins (Figures 2 and 3). While the CT TAP had excluded any major endoluminal thrombus of the main pulmonary arteries, it did not rule out a subsegmental PE. As he had had many CTPAs in the past, and was to be started on therapeutic dose clexane, a lung perfusion scan was performed to limit radiation exposure. This showed a single small subsegmental perfusion defect suggesting PE (Figure 4).

**Figure 5.**
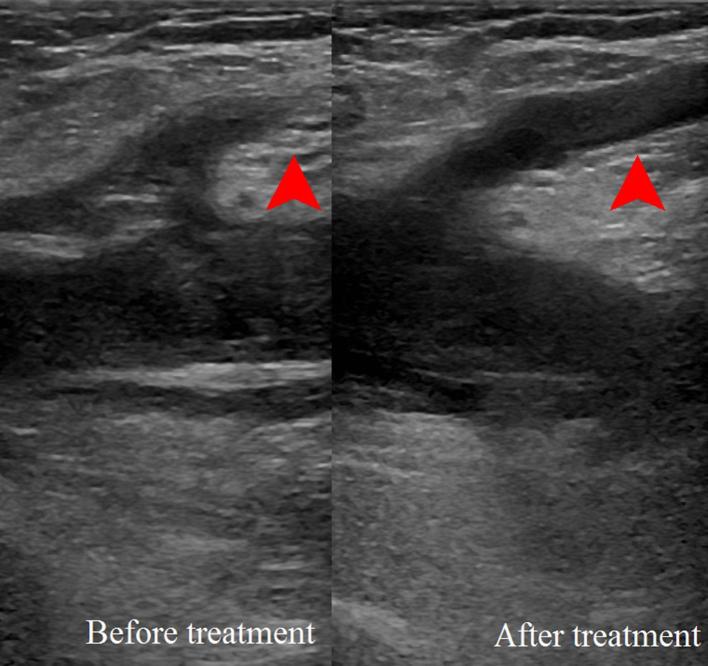
Ultrasound scan showing improvement in the right saphenofemoral junction.

He began treatment with 120 mg clexane. A follow-up ultrasound was performed 3 days later, which showed significant improvement but not complete resolution (Figure 5). He soon travelled back to New Zealand, against medical advice, and has since been lost to follow up.

## Discussion

Calcification in the IVC is an uncommon finding in adult populations.^7^ It is, however, more commonly seen in paediatric populations, and was first reported in 1961.^8^ A review of the literature in PubMed showed 26 cases in paediatric patients, and 12 in adults. The radiologically distinctive “bullet shaped” thrombus was described by Silverman et al in 1969, and is a triangular shaped calcified thrombus of the IVC found in children.^6^ This is usually asymptomatic and thus an incidental finding; it may not be unreasonable to postulate that the incidence in adults is actually higher than seen in the literature.^9^

Despite Morgagni describing the condition at post-mortem in 1769, the exact aetiology is still unclear.^5, 6^ Abdominal malignancy, structural abnormalities, coagulopathy, infection and compromised haemodynamic status have all been implicated.^5, 10^ It has also been described in association with antiphospholipid antibody syndrome by Cantisani et al.^11^

Kareem et al reported a case of a 23-year-old hypothyroid female, who had an IVC calcification which extended into her right atrium, for which no haematological or morphological cause was found.^10^ The authors speculate that the calcification may have extended into the right atrium along a pre-existing abnormal Eustachian valve.^10^ She was started on long term anticoagulant therapy.^10^

The only known case report which links IVC calcification to PE is by Chetwood et al, who presented a similar case of a 49-year-old male patient. In this case, like ours, the IVC calcification was discovered only after a recurring episode of PE.^5^ The authors agree with the conclusion of Chetwood et al that IVC calcification should be considered as a possible cause in patients with recurrent PE.^5^

Several authors have reported a correlation between IVC anomalies, mainly congenital such as atresia or hypoplasia, and recurrent DVT in young adults.^1, 3^ The basis of this correlation lies in the alteration of the haemodynamics of blood flow due to a disruption of the diameter and regularity of the vessel walls (Poiseuille’s Law).

As for the congenital abnormalities of the IVC, it is our suggestion that in the diffuse calcification of the IVC, the vessel is reduced in caliper and loses elasticity. This will inevitably increase the resistance to blood flow in the IVC. A distal increase in resistances will generate a proximal stasis of the blood. Eventually, this may even lead to endothelial injury. This will, as per Virchow’s Triad, increase the risk of clot formation.

At the time of the CT scan, our patient was on anticoagulant therapy, albeit an insufficient dose for an unknown reason. There was extensive thrombosis of the Iliac and femoral veins, but there were no clear sign of IVC thrombosis. We cannot exclude, however, that the clot formation of the previous DVT had started in the IVC and then progressed to the iliac and femoral veins.

CT is possibly the single best tool in the investigation of this condition, especially because it can exclude other common causes of DVT/PE. CT should be performed in cases of recurrent DVT and/or PE, and it is important that the radiologist pays particular attention to the IVC.

To confirm our hypothesis, a statistical correlation should be proven between unprovoked DVT/PE and IVC calcification, however, the cases of IVC calcifications are rare, and gathering a significant number will be challenging.

Long term anticoagulation alone has so far proven to be sufficient in managing this condition, but surgical intervention should be considered on an individual basis, especially in patients who cannot be effectively managed on anticoagulant therapy.

## Conclusion

IVC anomalies have been implicated in the pathogenesis of DVT. We hypothesise that IVC calcification similarly predisposes to DVT by interfering with venous return and predisposing to clot formation. IVC calcification is also theorised to predispose to PE.

In cases of DVT/PE of unknown cause, IVC calcification should be considered as a differential. Though rare, an awareness of IVC calcification may lead to faster diagnosis and management. Patients with this condition often require long-term anticoagulant therapy; therefore, delayed treatment may cause greater morbidity and mortality.

We suggest CT as the imaging modality of choice, and long-term anticoagulation as first line treatment. Surgical intervention should be considered on an individual basis.

## Learning points

1. IVC calcification is a rare cause of recurrent DVT & PE.
2. The investigation of choice for this condition is CT.
3. Long-term anticoagulation is usually sufficient to manage IVC calcification.
